# Supplementary material for: Chromothripsis during telomere crisis is independent of NHEJ, and consistent with a replicative origin
Source: Genome Res. 2019 May;29(5):737–49. doi: 10.1101/gr.240705.118 (PMC6499312; doi:10.1101/gr.240705.118)
Supplement: Supplemental Material [file supp_gr.240705.118_Supplemental_file_1.zip › contigs/annotated_contigs/DB114/contig.2.DB114_length_664_mean_cov_10.6174698795.docx]

**DB114_length_664_mean_cov_10.6174698795**

```
CTTCCCCTTCCACCATAATTCTACCTTTCCTGAGACCTCCCCAGCCATGCTGAACTGTGAGTCAATGAAACCTCTTTCTTCCATAAATT
 >chr16:83042167-83042526 - E=3e-204 p=3e-02
ACTCAGTCTTGGGCATGTCTTTATTAGCAGTGTCAGAATGGACTAATATAGAATAATCTGTGAAGATGAAAGGAAGGAGGGAGAAAGAG

AGGAAGGCCTCGTTCTGGCACAGGCCACCATGATCTTCTGCCTGGGTTACGGTGGTAAGCTCCTCACTGATTTCCCAATCCCTCATCCA

CCCATCTCCCACACCCAGTGGTCTCAACACAGCAAAGGTCCAAGTATAACATTATGTTTGTGTCACTCGTCTACTGAAAACCACCTAAT

T|AG|CTTCTGTTTTTGTAGCATTCTTTATTACAGCTGTTAGCCAATATCCTAACTGATTTGGATGCCCAGGTCTGAGCTGGCATAGTG
  >chr16:83037702-83038009 - E=6e-173
TTTTGTGAATTCAAAGTTCTTCCTTTTGAGCCTCCAGAGCCTTTGGCAGCATTCTATGACCCTTTCCTCCTGCTTCCCTATCTTTATCC

CTGACTCCTACAAAACCCGCTGCAAGGGAACACAGATACTCACGGTATAAGGAATGGGATTCAGCTGGCCTGGGGCTGTAGGTTTCTGC

TGGTCGGACTCAGTATTCTACAGTTGCATTCATTAACCCAAGA
```
